# Efficacy analysis of percutaneous pedicle screw fixation combined with percutaneous vertebroplasty in the treatment of osteoporotic vertebral compression fractures with kyphosis

**DOI:** 10.1186/s13018-020-1583-1

**Published:** 2020-02-17

**Authors:** Zhikun Li, Yi Wang, Youjia Xu, Wei Xu, Xiaodong Zhu, Chao Chen

**Affiliations:** 1 Department of Orthopedics, The Second Affiliated Hospital of Soochow University, 1055 Sanxiang Road, Suzhou, 215031 Jiangsu People’s Republic of China; 2 Tongren Hospital, School of Medicine, Shanghai Jiao Tong University, 1111 XianXia Road, Shanghai, 200336 People’s Republic of China

**Keywords:** PPSF, PVP, OVCF, Kyphosis

## Abstract

**Background:**

To investigate the clinical effect of percutaneous pedicle screw fixation (PPSF) combined with percutaneous vertebroplasty (PVP) in the treatment of osteoporotic compression vertebral fracture (OVCF) of the thoracolumbar vertebra with kyphosis.

**Methods:**

One hundred sixty-six patients before June 2017 were retrospectively analyzed, and patients were divided into PPSF + PVP group A and PVP group B. Operative time, bone mineral density, postoperative bed time, high compression ratio, bone cement leakage rate, and bone cement dose were recorded. Comparison of vertebral anterior edge height, Cobb angle, visual analogue score (VAS), and low back pain dysfunction index (ODI) between the two groups in preoperative, postoperative 3 days, postoperative 6 months, postoperative 12 months, and postoperative 24 months, postoperative complications were observed in the two groups.

**Results:**

The operation time of group A was longer than that of group B (59.0 ± 8.6 min, 26.6 ± 5.2 min), longer postoperative bed rest time (3.3 ± 0.7 days, 1.2 ± 0.5 days), the differences were statistically significant (*P* < 0.01), there was no difference in the amount of bone cement between the two groups (5.4 ± 0.6 ml, 5.3 ± 0.8 ml) (*P* > 0.05). The height of the anterior edge and Cobb angle of the two groups recovered significantly in postoperative 3 days. The height of anterior edge (2.7 ± 0.3 cm, 2.6 ± 0.2 cm, 2.5 ± 0.7 cm; 2.3 ± 0.6 cm, 1.7 ± 0.5 cm, 1.6 ± 0.3 cm) and Cobb angle (4.9 ± 2.2, 5.5 ± 2.3, 5.7 ± 2.3; 12.4 ± 3.2, 17.2 ± 2.5, 13.2 ± 2.3) was statistically significant in postoperative 6 months, postoperative 12 months, and postoperative 24 months (*P* < 0.01). VAS and ODI scores of postoperative 6 months and 12 months were significantly different between the two groups (*P* < 0.05). Postoperative complications in group B were much higher than those in group A.

**Conclusion:**

The efficacy of PVP alone was not satisfactory, and the rate of complications was high for OVCF patients with severe anterior edge compression with kyphosis. PPSF combined with PVP is recommended, the vertebral height loss was not obvious, the satisfaction was good, and the complication rate was lower during 2 years follow-up.

## Background

As the global aging process continues to accelerate, the number of osteoporosis patients increases, the most serious complication of osteoporosis is osteoporotic fracture, and the incidence of spinal fractures is the highest [1]. Percutaneous vertebroplasty (PVP) has been widely used to treat osteoporotic vertebral compression fractures (OVCF) for its rapid and effective relief of pain and enhancement of injured vertebrae [2, 3]. Polymethyl methacrylate (PMMA) is the most widely used bone cement material. Due to its significantly higher compression strength than osteoporotic bone and the uneven diffusion of bone cement, it is easy to cause the secondary fracture and collapse of injured vertebra after PVP, especially for the cases of “vacuum sign,” “effusion sign,” and “intravertebral cleft.” There is even a risk of compression of the spinal cord, and these can cause great harm to patients [4, 5].

Osteoporotic vertebral bodies are characterized by the reduction of trabecular bone and the widening of intervertebral spaces, and the bone structure in the vertebra is blurred. Minor trauma, such as falling down, carrying things, and coughing, can result in loss of vertebral height. This fracture can be reduced early (within 2 weeks) to restore vertebral height. The reduction methods include postural reduction, manipulative reduction, and distraction reduction of bone cement. Good vertebral height can be restored, and the wedge angle of the affected vertebra can be reduced. However, it has been reported in the literature that severe vertebral collapse, adjacent vertebral fracture, spinal instability, and other complications occurred during follow-up. In order to make up for the defects of vertebral collapse, adjacent vertebral fracture and spinal instability after PVP [6, 7]. PVP combined with percutaneous pedicle screw fixation (percutaneous pedicle screw fixation, PPSF) technology is used for strengthening and maintaining injured vertebral height, in order to reduce the occurrence of complications [8, 9]. However, there is a lack of literature on vertebral body weight compression with kyphosis, and the follow-up time in existing literature is short. In this study, patients with vertebral body weight compression with kyphosis were studied. The long-term clinical efficacy of PPSF combined with PVP and PVP alone was compared, and the objective was to summarize clinical experience and explore the advantages of PPSF combined with PVP in the treatment of OVCF with kyphosis.

## Methods

The study was approved by the ethics committee. A total of 166 cases were selected according to the EpiCalc 2000 software (Version 1.02), 83 cases of PPSF combined with PVP were treated in group A, and 83 cases of PVP were treated in group B.

### The research object

#### Inclusion criteria

(1) It was consistent with the diagnosis of osteoporotic vertebral fracture, with bone mineral density (BMD) *T* < − 2.5 SD. The image of fat inhibition in thoracolumbar MRI showed high signal. This is a fresh fracture. (2) The injured vertebra is a single segmental fracture of T11-L2. (3) The injured vertebra Cobb angle > 10° and belongs to Genant III, that is, the height difference between the anterior and posterior height of the vertebra is more than 40%. (4) Physical examination showed no nerve injury. CT showed no obvious spinal canal occupation. (5) The age ranges from 60 to 90 years. (6) Complete imaging data are available, including preoperative and postoperative X-rays. Follow-up X-ray was performed at 6 months, 12 months, and 24 months after operation.

#### Exclusion criteria

(1) There are absolute surgical contraindications, such as the patients combined with serious cardiopulmonary function and coagulation dysfunction. The base condition is too poor to tolerate surgery; (2) non-osteoporotic fractures and pathologic fractures caused by other causes, such as tumor destruction and spinal infection; (3) old OVCF; (4) patients who have neurologic symptoms on physical examination or the patients who need spinal decompression surgery as the CT show intraspinal space has been occupied; (5) BMD > − 2.5 SD or BMD < − 5 SD; (6) multiple segmental fractures; and (7) incomplete image data.

### Therapy

1. *Group A* (*PPSF combined with PVP*). The patient lies prone on the spinal surgery bed after general anesthesia. Manipulative reduction was performed at the beginning of the operation. First step (PPSF): After G-arm fluoroscopy, the projection position of the injured vertebral pedicle and adjacent segments was marked. After disinfection, 4 longitudinal incisions of approximately 1.5 cm were made at the mark, exposing the screw placement position “Herringbone spines” of the vertebral body through the multifissure gap. Four pedicle screws were inserted with fluoroscopic guidance at the appropriate angle. Second step (PVP): At the location of the body surface of the injured vertebral pedicle, the puncture needle should maintain a certain abduction angle and rake angle to enter the vertebral pedicle. Lateral fluoroscopy showed that the puncture stopped at the junction of the anterior middle 1/3 of the vertebra. Pull out the core of the puncture needle and start preparing PMMA bone cement. Bone cement was injected under the monitoring of G-arm during the “wiredrawing stage,” and the dispersion of bone cement in the vertebral body was closely observed. Before the bone cement was dispersed to the posterior edge of the vertebral body, the injection was stopped, and no leakage of vertebral canal or intervertebral foramen was confirmed. The total amount of bone cement injected into the T11 and T12 vertebral bodies was about 3‑5 ml, and the L1 and L2 vertebral bodies were about 5‑6 ml. Remove the needle after the cement has completely set for about 10 min. Finally, select the appropriate length of the connecting rod, install the double connecting rod, and tighten the nut. Both PPSF and PVP systems are provided by Stryker.
2. *Group B* (*PVP*). The patient lies prone on the spinal surgery bed. After local anesthesia, fracture reduction by manipulation was performed routinely. The position of the injured pedicle was located and marked under G-arm fluoroscopy. PVP was treated in the same way as group A.
3. *Postoperative treatment*. Patients in both groups were given oral calcium carbonate D3 and ossified triol and intravenous zoledronic acid for anti-osteoporosis treatment. Ask the patient to lie supine for 2 h after surgery to compress the puncture wound. Patients are encouraged to exercise the lumbar dorsal muscle function and lift the leg in bed at the early stage. Generally, patients in group A could get out of bed on the third day after surgery, while patients in group B could get out of bed on the first day after surgery. Both groups of patients had rehabilitation physicians to guide the lumbar and back functional exercise and out of bed movement exercise. It is suggested that the patient should go to the clinic for a bone mineral density review every year after surgery.

### Research data

#### General situation

The general situations are as follows: operation time, bone mineral density *T* value, bone cement injection amount, and bone cement leakage.

#### Imaging

Preoperative, postoperative 3 days, postoperative 6 months, postoperative 12 months, and postoperative 24 months all received X-ray examination. The percentage of compression of the anterior height of the injured vertebra and the posterior convex angle of the sagittal vertebras (Cobb angle) were measured and calculated on the lateral X-ray. The percentage of compression of the anterior height of the injured vertebra = (posterior height of the injured vertebra − anterior height of the injured vertebra)/posterior height of the injured vertebra× 100%. Cobb angle is the included angle between the lower endplate of the upper segment of the injured vertebra and the upper endplate of the next segment of the injured vertebra.

#### Clinical evaluation

Visual analogue scale (VAS) was used to score the pain degree. The Oswestry Disability Index (ODI) was used to evaluate the degree of disability in daily activities and to observe the complications in perioperative period.

### Statistical analysis

The SPSS18.0 statistical software was used for statistical analysis. Quantitative data are expressed as mean ± standard deviation. The gender ratio and segment of the injured vertebra were compared by chi-square test. Age, bone mineral density *T* value, operation time, amount of injured vertebra cement, postoperative bed time, percentage of injured vertebra anterior height, Cobb angle, VAS score, ODI score, and other indicators were compared by independent sample *t* test. Within the group, the measurement parameters were compared between the same period before surgery, 3 days after surgery, 6 months after surgery, 12 months after surgery, and 24 months after surgery by paired *t* test. *P* < 0.05 indicated that the difference was statistically significant.

## Results

### General situation

Review the patients with OVCF who received surgical treatment in our hospital before June 2017. One hundred sixty-six patients met the inclusion criteria and were followed up for at least 2 years. According to different surgical methods, the patients were divided into group A and group B, and there was no statistical difference in general preoperative information between the two groups (*P* > 0.05). In group A, the operation time was 59.0 ± 8.6 min, the bone cement content of the injured vertebrae was 5.4 ± 0.6 ml, and the postoperative bed time was 3.3 ± 0.7 days. In group B, the operation time was 26.6 ± 5.2 min, the bone cement content of the injured vertebrae was 5.3 ± 0.8 ml, and the postoperative bed time was 1.2 ± 0.5 days. Compared with the only PVP treatment in group B, patients in group A had a statistically significant difference in operation time and postoperative bed time (*P* < 0.01). There was no difference in the bone cement leakage, the ratio of preoperative vertebral height compression, and the amount of bone cement between the two groups (*P* > 0.05) (Table 1).

**Table 1:** Comparison of different surgical methods for the treatment of OVCF with kyphosis

	Group A	Group B	Test value	*P*
Number	83	83		
Age (year)	73.6 ± 11.0	75.5 ± 10.6	*T* = − 1.094	0.275 > 0.05
Gender	16 male/64 female	15 male/83 female	*X*^2^ = 0.675	0.411 > 0.05
T11	14	12	*X*^2^ = 0.511	0.916 > 0.05
T12	29	28		
L1	24	28		
L2	16	15		
Pre-*T* value	− 3.1 ± 0.3	− 3.1 ± 0.4	*T* = − 0.56	0.576 > 0.05
Post 1 year-*T* value	− 3.2 ± 0.4	− 3.0 ± 0.3	*T* = − 1.699	0.097
Post 2 year-*T* value	− 3.1 ± 0.3	− 3.2 ± 0.5	*T* = − 0.178	0.859
Operation time (min)	59.0 ± 8.6	26.6 ± 5.2	*T* = 29.26	0.000 < 0.01
Amount of bone cement (ml)	5.4 ± 0.6	5.3 ± 0.8	*T* = 0.335	0.738 > 0.05
Postoperative bed time (h)	3.3 ± 0.7	1.2 ± 0.5	*T* = 22.501	0.000 < 0.01
Bone cement leakage rate (%)	17.8 (14)	14.3 (12)	*X*^2^ = 0.182	0.669 > 0.05
Height compression rate (%)	53.6 ± 12.1	50.9 ± 10.2	1.54	0.125 > 0.05

### Imaging

The anterior height of the injured vertebra in group A was restored from 1.3 ± 0.4 cm before surgery to 2.8 ± 0.2 cm 3 days after surgery, 2.7 ± 0.3 cm 6 months after surgery, 2.6 ± 0.2 cm 1 year after surgery, and 2.5 ± 0.7 cm 2 years after surgery. The Cobb angle of the injured vertebra recovered from 23.1 ± 5.8° before surgery to 5.6 ± 2.6° 3 days after surgery, 4.9±2.2° 6 months after surgery, 5.5±2.3° 1 year after surgery, and 5.7±2.3° 2 years after surgery.

The anterior height of the injured vertebra in group B was restored from 1.2 ± 0.5 cm before surgery to 2.7 ± 0.3 cm 3 days after surgery, 2.3 ± 0.6 cm 6 months after surgery, 1.7 ± 0.5 cm 1 year after surgery, and 1.6 ± 0.3 cm 2 years after surgery. The Cobb angle of the injured vertebra recovered from 24.0 ± 5.4° before surgery to 5.1 ± 2.0° 3 days after surgery, 12.4 ± 3.2° 6 months after surgery, 17.2 ± 2.5°1 year after surgery, and 13.2 ± 2.3° 2 years after surgery.

**Fig. 1:**
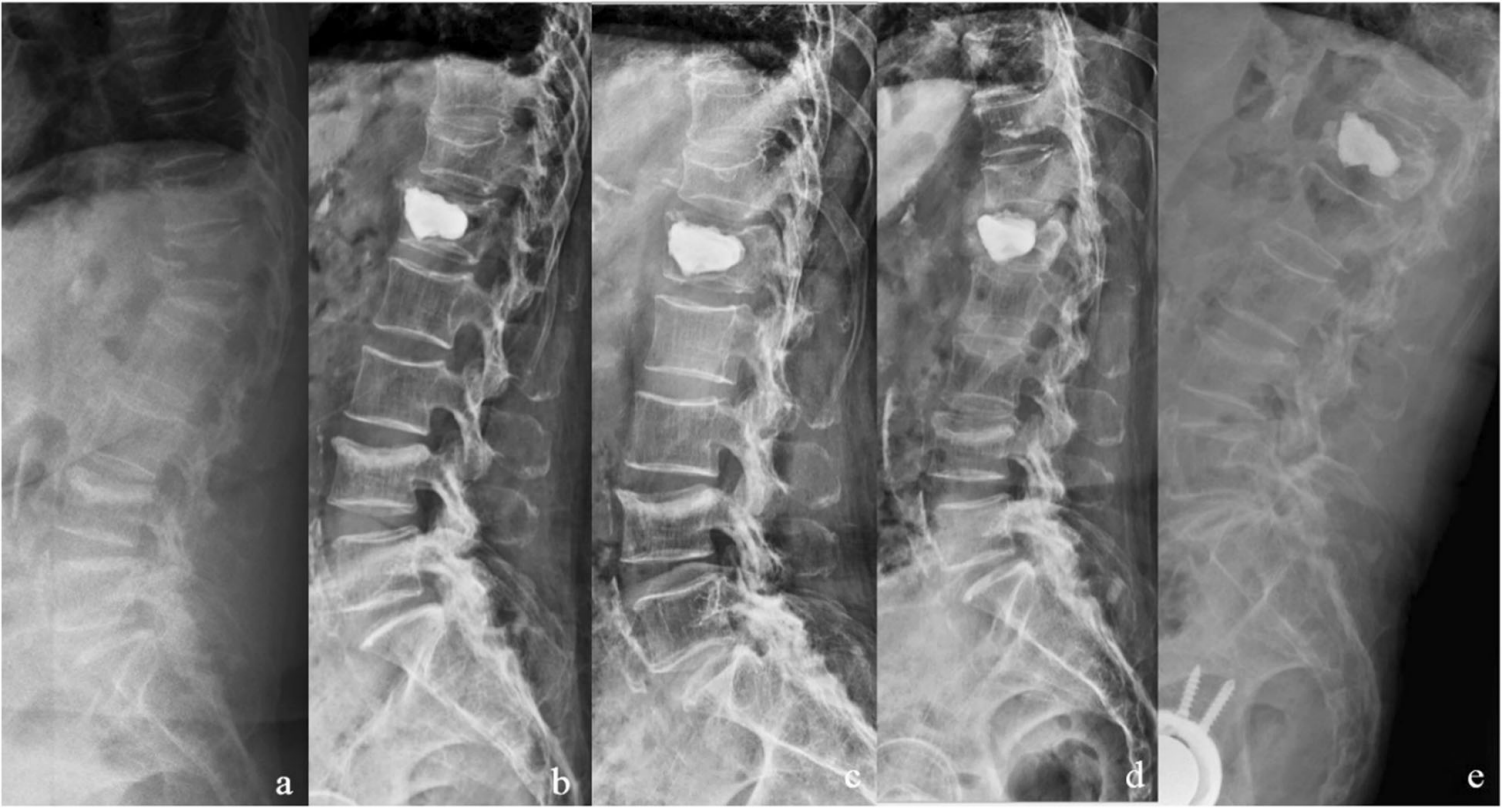
A 73-year-old female patient with osteoporotic compression fracture of L1 vertebral body with kyphosis was treated with PVP alone. **a** L1 severe compression fracture with kyphosis in preoperative. **b** Postoperative 3D X-ray showed that the height of L1 vertebra recovered basically and kyphosis was corrected. **c**–**e** X-ray showed L1 vertebral was collapsed with kyphosis in 6 months, 12 months, and 24 months follow-up

The measurement results of imaging before and after the surgery were analyzed as follows: (1) Comparison between the two groups—there were no significant differences in the anterior height and Cobb angle of the injured vertebra between the two groups before and after 3 days (*P* > 0.05). However, there were significant differences in the anterior height and Cobb angle of the injured vertebra between the two groups at 6 months, 12 months, and 24 months postoperatively (*P* < 0.01). (2) Comparison in intra-group—the anterior height and Cobb angle of the injured vertebra in the two groups recovered significantly after 3 days operation compared with that before operation. There were no significant differences in the anterior height and Cobb angle of the injured vertebra in group A 3 days after surgery, 6 months after surgery, 12 months after surgery, and 24 months after surgery (*P* > 0.05). During the postoperative follow-up, there were no cases of the injured vertebra collapsing again. There were significant differences in the anterior height and the Cobb angle of the injured vertebra in group B 3 days after surgery, 6 months after surgery, 12 months after surgery, and 24 months after surgery (*P* < 0.05). All patients gradually developed anterior collapse of the injured vertebra after surgery. The anterior height of the injured vertebra was most seriously lost in 6 months after surgery (Table 2 and Fig. 1).

**Table 2:** Comparison of different surgical methods for the height of anterior edge and the Cobb angle

Group	Number	The anterior height of injured vertebra (cm)					Cobb angle of injured vertebra (°)				
		Pre-	Post-3 days	Post-6 months	Post-12 months	Post-24 months	Pre-	Post-3 days	Post-6 months	Post-12 months	Post-24 months
A	83	1.3 ± 0.4	2.8 ± 0.2^①^	2.7 ± 0.3^②^	2.6 ± 0.2^②^	2.5 ± 0.7^②^	23.1 ± 5.8	5.6 ± 2.6^①^	4.9 ± 2.2^②^	5.5 ± 2.3^②^	5.7 ± 2.3^②^
B	83	1.2 ± 0.5	2.7 ± 0.3^①^	2.3 ± 0.6^①^	1.7 ± 0.5^①^	1.6 ± 0.3^①^	24.0 ± 5.4	5.1 ± 2.0^①^	12.4 ± 3.2^①^	17.2 ± 2.5^①^	13.2 ± 2.3^①^
T	–	1.270	1.152	10.607	21.581	26.272	− 1.120	1.501	− 17.632	− 30.889	− 20.916
P	–	> 0.05	> 0.05	< 0.01	< 0.01	< 0.01	> 0.05	> 0.05	< 0.01	< 0.01	< 0.01

^①^There are significant differences with the previous data, *P* < 0.05

^②^There is no significant difference from the previous data, *P* > 0.05

### Clinical evaluation

#### Satisfaction

*Comparison of satisfaction between the two groups*. VAS and ODI scores of the two groups before surgery, 3 days after surgery, and 24 months after surgery showed no significant difference (*P* > 0.05). VAS and ODI scores at 6 months and 12 months after surgery were significantly different between the two groups (*P* < 0.05).

**Fig. 2:**
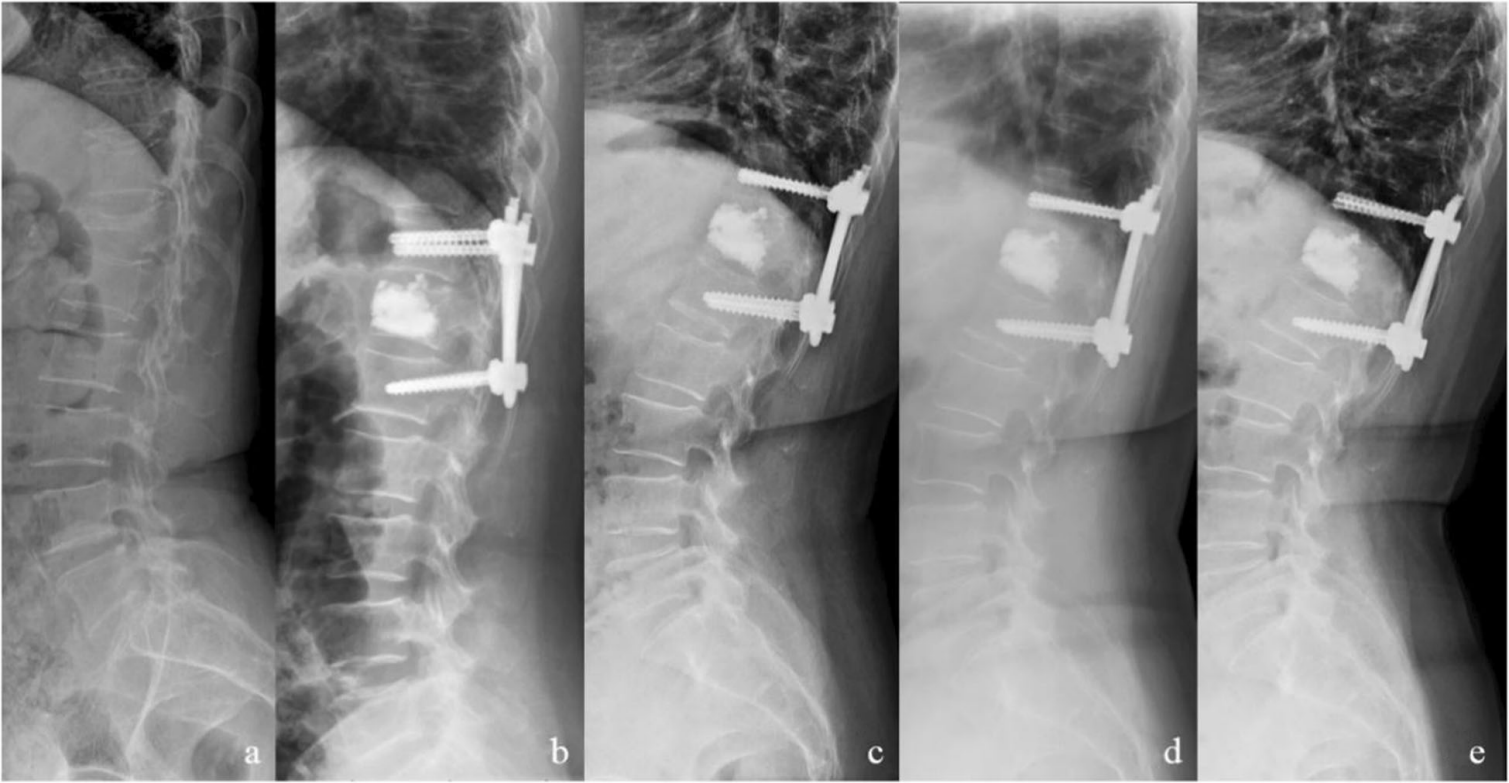
A 71-year-old female patient with osteoporotic compression fracture of T12 vertebral body with kyphosis was treated with PPSF combined with PVP. **a** T12 severe compression fracture with kyphosis in preoperative. **b** Postoperative 3D X-ray showed that the height of L1 vertebra recovered basically and kyphosis was corrected. **c**–**e** X-ray showed no significant loss of T12 vertebral body height and kyphosis in 6 months, 12 months, and 24 months follow-up

*Comparison of satisfaction at different follow-up periods in the group*. Postoperative VAS score and ODI score of the two groups were significantly lower than those before the operation (*P* < 0.05). VAS and ODI of group A at post-6 months, post-12 months, and post-24 months showed no statistical difference (*P* > 0.05). There were statistical differences in VAS and ODI between post-6 months, post-12 months, and post-24 months in group B (*P* < 0.05) (Table 3 and Fig. 2).

**Table 3:** Comparison of different surgical methods for VAS and ODI

Group	Number	VAS					ODI				
		Pre-	Post-3 days	Post-6 months	Post-12 months	Post-24 months	Pre-	Post-3 days	Post-6 months	Post-12 months	Post-24 months
A	83	7.8 ± 0.6	2.5 ± 0.6^①^	2.5 ± 0.7^②^	2.5 ± 0.6^②^	2.8 ± 0.6^②^	82.5 ± 5.3	31.5 ± 3.2^①^	28.5 ± 3.2^②^	25.8 ± 3.0^②^	25.4 ± 2.2^②^
B	83	7.7 ± 0.7	2.6 ± 0.7^①^	3.3 ± 0.8^①^	3.8 ± 0.9^①^	3.0 ± 0.7^①^	83.8 ± 4.9	30.8 ± 3.3^①^	36.6 ± 4.8^①^	32.2 ± 4.3^①^	26.1 ± 4.0^①^
T	–	1.030	− 0.962	− 6.389	−10.532	− 0.685	− 1.639	1.369	− 12.989	− 10.981	− 1.406
P	–	0.304	0.338	< 0.001	< 0.001	0.494	0.103	0.173	< 0.001	< 0.001	0.162

^①^There are significant differences with the previous data, *P* < 0.05

^②^There is no significant difference from the previous data, *P* > 0.05

#### Complication

There were 14 cases of bone cement leakage in group A (17.8%) and 12 cases of bone cement leakage in group B (14.3%). There was no bone cement leakage in the intraspinal and intervertebral foramina in both groups, and no nerve injury was found. The two groups of patients did not see bone cement venous leakage and pulmonary embolism phenomenon. In group A, there was no significant vertebral height loss or kyphosis, and 1 case of lumbago and back pain due to loose internal fixation was found. The symptoms were relieved after removal of the cement screw after internal fixation. All the patients in group B had obvious vertebral height loss and kyphosis (Cobb angle greater than 10°) during the 6 months follow-up after the operation, among which 15 cases (18.1%) had secondary spinal stenosis with paraplegia, 5 cases had remission of lower limb paraplegia after conservative treatment, 10 cases were treated with posterior lumbar decompression and internal fixation, among which 8 cases had postoperative relief of lower limb insufficiency, and 2 cases had lower limb muscle strength of grade 2–3 and were unable to walk and stay in bed for a long time. There were 5 cases (6.0%) in group A and 1 case (1.2%) in group B. No wound infection was found in either group.

## Discussion

### Results of this study

In this study, patients were divided into two groups according to different surgical methods for retrospective study. PPSF combined with PVP was used in group A, and PVP alone was used in group B. There was no significant difference in the postoperative vertebral anterior height (2.8 ± 0.2, 2.7 ± 0.3), Cobb angle (5.6 ± 2.6, 5.1 ± 2.0), VAS (2.5 ± 0.6, 2.6 ± 0.7), and ODI (31.5 ± 3.2, 30.8 ± 3.3) (*P* > 0.05). This suggests that short-term outcomes are similar. However, during follow-up at 6 months, 12 months, and 24 months after surgery, the vertebral anterior height in group B decreased (2.3 ± 0.6, 1.7 ± 0.5, 1.6 ± 0.3), and the Cobb angle increased (12.4 ± 3.2, 17.2 ± 2.5, 13.2 ± 2.3). There were significant differences (*P* < 0.05) between the vertebral anterior height in group A (2.7 ± 0.3, 2.6 ± 0.2, 2.5 ± 0.7) and the Cobb angle (4.9 ± 2.2, 5.5 ± 2.3, 5.7 ± 2.3) (*P* < 0.05). This indicates that PPSF combined with PVP can maintain the anterior height for a long time after surgery. VAS (3.3 ± 0.8, 3.8 ± 0.9) and ODI (36.6 ± 4.8, 32.2 ± 4.3) were increased in group B at 6 months and 12 months after surgery, and VAS (2.5 ± 0.7, 2.5 ± 0.6) and ODI (28.5 ± 3.2, 25.8 ± 3.0) were compared with those in group A at the same period, showing statistical significance (*P* < 0.05). At 24 months after surgery, VAS (3.0 ± 0.7) and ODI (26.1 ± 4.0) scores in group B were decreased again, and VAS (2.8 ± 0.6) and ODI (25.4 ± 2.2) in the same period were not significantly different from those in group A (*P* > 0.05). The reason is that after the operation, another fracture of the injured vertebra occurred at 6 months, and the vertebral height was lost too fast, leading to the aggravation of the patient’s back pain and the dysfunction of the lumbar movement. PPSF combined with PVP reduced the loss of vertebral height. In group A, the study found that there was no significant difference in the anterior height and the Cobb angle during the 2-year follow-up compared with the 3-day postoperative comparison, and the VAS and ODI scores of the patients were stable. Bone cement leakage (17.8%, 14.3%) and subcutaneous hematoma (2 cases, 1 case) occurred in both groups, but there was no significant difference between the two groups (*P* > 0.05). All patients in group B showed kyphosis at 6 months of postoperative follow-up, while patients in group A were followed up for 2 years without kyphosis. In group B, 15 patients had serious postoperative complications: secondary spinal stenosis with incomplete paralysis, with an incidence of 18.1%. One case of internal fixation loosening occurred in group A, indicating that the incidence and severity of complications in group B were higher than those in group A. Therefore, in this study, it was suggested that OVCF patients with severe compression and kyphosis should not be treated with PVP alone, but with PPSF combined with PVP.

### Significance and phenomenon analysis of this study

With the development of medical technology, the pointer of PVP surgery has become wider and wider, and many patients have achieved satisfactory results, but many problems have also emerged, including vertebral height loss, kyphosis, secondary fracture, secondary vertebral fracture, nerve compression, and revision surgery. There have been many studies on the use of PVP for OVCF with severe compression [10], but all of them lack long-term follow-up. This study found that for OVCF patients with kyphosis, the use of PVP alone can achieve good vertebral height reduction after surgery. However, at 6 months after the operation, significant height loss, increased Cobb angle, loosening of bone cement, aggravation of low back pain, and increase of VAS and ODI scores began to occur, those indicating unsatisfactory treatment effect, and indicating that OVCF with severe compression and kyphosis may not be suitable for PVP treatment. However, it was found that PPSF combined with PVP could significantly reduce the incidence of height loss, kyphosis, and other complications.

OVCF with kyphosis associated with severe compression refers to the case with anterior height reduction of more than 40% and Cobb angle greater than 10°. Osteoporosis is characterized by sparse vertebral trabeculae, the anterior height of the severely compressed vertebral body recovered after reduction. However, a cavity, called a “vacuum zone,” forms within the vertebra, which is a cavity similar to intravertebral cleft, vacuum, or effusion. If PVP treatment results in the formation of lumpy bone cement and poor dispersion, the study found that this form of bone cement leads to more stress concentration [11]. Compared with conventional PVP-injected bone cement with good dispersion, this mass of bone cement in the osteoporotic vertebral body is similar to “date pit” in tofu. The strength of bone cement is much stronger than that of human bone, and such patients will quickly lose vertebral height, or secondary vertebral fractures, after PVP treatment. Therefore, we suggest that OVCF with severe compression and kyphosis should not be treated with PVP alone.

### Compared with previous studies

Earlier studies have shown that vertebral augmentation (PVP\PKP) can be used to treat OVF with severe compression. Lee et al. [12] found that after PKP treatment, the anterior height recovered from 6.4 ± 2.1 mm before surgery to 17.2 ± 4.4 mm after surgery, and the height compression ratio recovered from 24.0 ± 6.4% before surgery to 66.3 ± 14.9% after surgery. PKP significantly accelerated pain recovery and restored the anterior height, and they are suggesting that PKP is a reliable method for patients with severe compression of OVF. The research of Sun et al. [13]and Kim et al. [14] also agrees with this view. But then, something went wrong. Kim et al. [15] believed that progressive vertebral height loss would occur after the treatment of OVF with vertebral augmentation, which was inevitable. The cases after PKP were particularly serious, which might be the result of different contact between bone cement surfaces in PKP balloons. There are many reasons for vertebral height loss after vertebral augmentation, for example, the severity of osteoporosis, the choice of surgical methods, the different surgical approaches, the different degrees of preoperative vertebral compression, the different distributions of bone cement, the different amounts of bone cement injection, or the existence of intravertebral cleft and other reasons [16–23]. Therefore, vertebroplasty alone is not appropriate for the treatment of OVF with severe compression and kyphosis.

The technique of PPSF combined with PVP was first reported in 2005 by Acosta et al. [24] for the treatment of traumatic lumbar burst fractures, and it was applied to the treatment of OVCF subsequently. Gu et al. [8] believed that PPSF combined with PVP is a better method for the treatment of acute OVCF and plays a role in the prevention of new VCF. Yang et al. [25] also reached the same conclusion from the perspective of biomechanics, and the results showed that in the treatment of OVCFs, short-segment PSF + PKP could reduce the fracture risk of treated vertebral bodies and adjacent untreated vertebral bodies. Wang et al. use PPSF + PVP treatment of thoracic lumbar osteoporotic compression fractures, and the results showed that PPSF combined with PVP could achieve stronger vertebral strength and stiffness than PVP alone in treating OVCF of thoracic and lumbar segments, which was more conducive to improving the reduction effect of injured vertebrae and maintaining the anterior height of injured vertebrae and preventing vertebral body collapse. However, there was no research report on OVCF with severe compression and kyphosis. According to Gu et al. [26], compared with OVCF with kyphosis treated by PVP alone, PPSF combined with PVP can achieve better recovery of the anterior height and Cobb angle. This may be related to the distraction and reduction of pedicle screw during surgery. However, the results of this study showed that there was no significant difference in the anterior height and Cobb angle between the two groups after the operation. Considering the presence of osteoporosis in the vertebral body, distraction and reduction was not performed during the PPSF operation in this study. In addition, the amount of bone cement used by PPSF combined with PVP in our study was not different from that of PVP alone, which was different from previous studies [9]. VAS and ODI scores began to show differences between the two groups at 6 months after surgery, which was also different from the previous literature [9]. VAS and ODI scores were also different between the two groups at 1 and 2 years after the operation, indicating that the long-term effect of PVP alone on OVCF with kyphosis was not good. This is different from the results of previous studies, which may be caused by different study subjects. The former study subjects were patients with mild vertebral compression, while the present study subjects were patients with severe compression with kyphosis.

### Deficiencies

First, the sample size of this study was estimated by the EpiCalc 2000 software (Version 1.02), which required 83 cases for each group, and the total sample size was 166 cases. As a retrospective study found that a large number of patients were lost to follow-up, the study had to use the minimum sample size. Second, for surgical patients who cannot tolerate general anesthesia, only PVP alone can be used for treatment. These patients who are over 90 years old are not included in the study, so there is a selection bias. Third, for patients with OVCF, it is more appropriate to use bone cement screws because the internal fixation of bone cement screws is more firm. However, the medical cost is much more expensive than ordinary screws, so we can only use ordinary screws in clinical work. However, during our 2-year follow-up, only 1 patient (1.2%) showed significant loosening of internal fixation and accompanied by symptoms of low back pain. We recommend the use of bone cement screws for patients with a *T* value < 4 SD. Fourth, at 1 year after the treatment of OVCF with PPSF combined with PVP, a small number of patients were removed with internal fixation. Follow-up revealed that vertebral height loss in patients with internal fixation was more serious than that in patients without internal fixation, but the sample size was too small and no further study was conducted.

### Expectation

The short-term effect of vertebral augmentation on OVCF is positive, but the biomechanical properties of bone cement (80 MPa) and human vertebral bone (10 MPa) are far different, so the biomechanical changes of the spine are bound to occur after bone cement implantation. There is an urgent need to develop a new type of bone cement that is similar in strength to the vertebral bone, which may alleviate or even solve these problems. But on the other hand, osteoporosis is the normal natural law of human aging, and the progress of medicine can delay aging. Do not go against the natural law.

## Conclusion

For OVCF patients with severe compression of the anterior height and kyphosis, both PPSF combined with PVP and PVP alone can achieve good recovery and satisfaction of the anterior height of the vertebral body in the short term after surgery. However, in the 2-year follow-up, all patients in the PVP group showed loss anterior height of injured vertebra and kyphosis at 6 months after the operation, with decreased satisfaction, and the complication rate was significantly higher than that in the PPSF combined PVP group. Therefore, for OVCF with severe compression and kyphosis, PPSF combined with PVP is recommended for treatment. During long-term follow-up, the height loss of vertebral reduction is not obvious, satisfaction is good, and the incidence of complications is lower.

## Data Availability

The datasets used and/or analyzed during the current study are available from the corresponding author upon reasonable request.

## Abbreviations

BMD: Bone mineral density
ODI: Oswestry Disability Index
OVCF: Osteoporotic vertebral compression fractures
PMMA: Polymethyl methacrylate
PPSF: Percutaneous pedicle screw fixation
PVP: Percutaneous vertebroplasty
VAS: Visual analogue scale
